# Aerodynamic performance and wind-induced response of carbon fiber-reinforced polymer (CFRP) cables

**DOI:** 10.1038/s41598-024-59002-w

**Published:** 2024-04-08

**Authors:** Guanxu Long, Yamin Sun, Zhiqiang Shang, Xiaoyu Wang, Xingwei Xu, Tao Wang

**Affiliations:** 1 Shandong Key Laboratory of Highway Technology and Safety Assessment, Jinan, 250000 Shandong China; 2 Shandong Hi-Speed Group Co. LTD Innovation Research Institute, Jinan, 250102 Shandong China; 3 School of Architecture and Civil Engineering, Xi’an University of Science and Technology, Xi’an, 710054 Shaanxi China; 4 Gezhouba Group Transportation Investment Co., LTD, Wuhan, 430030 Hubei China; 5 Shandong Hi-Speed Engineering Test CO., LTD, Jinan, 250000 Shandong China; 6 School of Highway, Chang’an University, Xi’an, 710064 Shaanxi China

**Keywords:** Engineering, Materials science

## Abstract

To study the aerodynamic performance and wind-induced response of carbon fiber-reinforced polymer (CFRP) cables, CFRP cable was designed by replacing a steel cable in a tied arch bridge based on stiffness, strength and area equivalent criteria, respectively. The aerodynamic performance and wind-induced response of CFRP cable and steel cable were studied and compared by computational fluid dynamics (CFD) model. Based on the computational results, optimal cable replacement criterion was proposed for CFRP cable to replace steel cable. In addition, surface modification was conducted by engrooving vertical symmetric (VS), vertical asymmetric (VA) and helical symmetric (HS) V-shaped grooves to improve the aerodynamic performance and wind-induced response of CFRP cable. Results showed that CFRP cables exhibited inferior aerodynamic performance and wind-induced response in most cases. However, CFRP cable based on stiffness equivalent criterion exhibited better aerodynamic performance and wind-induced vibration properties compared to the other two cable replacement criteria, thus is regarded as the optimal substitute for steel cable. In addition, HS grooves generated symmetric disturbances and caused approximately equivalent boundary layer separation delays uniformly and continuously along the cable length, thus exhibiting better effect in decreasing the reverse flow region, the maximum negative flow velocity, the vortex shedding frequency and the wind-induced vibration amplitude of CFRP cable. Hence, the stiffness equivalent criterion combined with surface modification with HS V-shaped grooves was proposed to replace steel cable with CFRP cable. This study can provide insights into the aerodynamic performance and wind-induced response of CFRP cable and instructions for cable replacement practice.

## Introduction

Carbon fiber-reinforced polymer (CFRP) has advantages such as high tensile strength, lightweight, outstanding corrosion and fatigue resistance. Among these advantages, the superior corrosion resistance makes CFRP popular in civil engineering and thus is regarded as an optimal substitute for traditional steel cables in bridge engineering to extend the maintenance period and to increase the span ability of cable supported bridges^1–3^. Researchers and engineers have proposed design proposals to cross straits or channels using cable supported bridges with CFRP cables^4–6^. Several demonstration bridges have also applied CFRP as cable system both in newly construction cases^7,8^ and rehabilitate projects^9,10^, showing the feasibility of adopting CFRP as cables in bridge sector.

Though CFRP cable has promising application prospect in cable supported bridges, some critical issues must be settled before it is widely used. One of the most critical issues is the aerodynamic and wind-induced response. Due to the low self-weight of CFRP cable, it is much more sensitive to wind load than steel cable. When CFRP cable is used in cable supported bridges, the wind-resistance stability of entire bridge is different from that of supported bridges with steel cables. Xiong et al.^11^ studied the wind-resistance stability of cable-stayed bridges with steel and CFRP cables and found that wind-resistance stability of cable-stayed bridge with CFRP and steel composite cables was inferior to that of cable-stayed bridge with steel cables, but was superior to that of cable-stayed bridge with CFRP cables. Bai et al.^12^ analyzed the flutter and buffeting characteristics and aerostatic stability of cable-stayed bridges with fiber-reinforced polymer (FRP) cables and girders and found that cable-stayed bridge incorporating FRP cables and girders exhibited poorer aerostatic stability compared to conventional steel cable-stayed bridges. Besides, the flutter and buffeting critical wind speeds of cable-stayed bridge with FRP cables were also lower than those of cable-stayed bridge with steel cables. This indicates that use of FRP cables has an obvious influence on the aerodynamic properties of the entire bridge and the aerodynamic properties of FRP cables also need to be studied. However, Zhang et al.^13^ and Yang et al.^14^ studied the critical wind speed of CFRP cable-supported bridge and steel cable-supported bridge and found that the critical wind speed of former pattern was larger than the latter one. The stark differences between the aforementioned research findings can be attributed to two factors: first and most important, current research on CFRP cable-supported bridges was based on steel cable-supported bridges by replacing steel cables with CFRP ones based on different cable replacement criterion. Literature^11^ adopted strength equivalent criterion while stiffness equivalent criterion was adopted in^12–14^. In addition, most studies on CFRP cable-supported bridges only replace steel cable with CFRP ones, while steel cables and girder were all replaced with CFRP ones in^11^.

As an important component and load-bearing system of cable-stayed bridges, the wind loads and wind-induced vibrations experienced by stay cables reflect the wind environmental characteristics imposed on the main girder and bridge deck. The aerodynamic performance of the cables directly influences the overall aerodynamic performance of the bridge. Astiz^15^ demonstrated that aeroelastic effects of cable system including conjunction between traffic, vortex shedding and buffeting were worthwhile to be studied as it happened frequently and contributed much to fatigue in the cables of cable-supported bridges. Hence, the aerodynamic response and wind-induced vibration of CFRP cables have close ties and interactions with the overall aerodynamic response of the entire CFRP cable-supported bridge and are necessary to be studied.

In terms of the aerodynamic properties of FRP cables, Chen et al.^16^ found though with identical cross-sectional area, CFRP and steel cables exhibited different aerodynamic performance. Yang et al.^17^ replaced steel cable with CFRP cable with strength equivalent criterion and compared the aerodynamic performance of steel and CFRP cables. Results showed that the wind pressure on the cable surface and the wind-induced vibration frequency for CFRP cable were all larger than those for steel cable. Wu et al.^18^ found that the vortex induced vibration amplitude and critical speed of wind-rain induce vibration of CFRP cable based on strength equivalent criterion were all larger than those of steel cable. Overall, there is currently limited research on the aerodynamic performance and wind vibration characteristics of CFRP cables. Moreover, existing studies only involve comparative analyses between CFRP and steel cables under specific cable replacement conditions.

As mentioned above, the aerodynamic performance and wind-induced response of CFRP cables are inferior to steel cables. Therefore, an important task for scholars and engineers is to mitigate the aerodynamic and wind-induced vibration response of CFRP cables. Installing dampers and setting wind rope can suppress the wind-induced vibration. However, these measures disrupt the aesthetics of the structure. Another effective way to improve the wind-induced vibration properties is aerodynamic configuration modification. Researchers have tried ways to modify the surface configuration of steel cables and achieved great success. Typical approaches involved altering cable surface roughness^19,20^, incorporating surface indentations^21,22^, helical or longitudinal ridges^22–26^, grooved textures^27^ and wire meshes^28–30^. However, the above efforts all concentrated on steel cables. As FRP cables are more flexible than steel cables, the aerodynamic and wind-induced response of CFRP cables are more obvious than those of steel cables. Hence, the mitigation methods for large aerodynamic and wind-induced response of CFRP cables also should be studied.

In this study, CFRP cables are designed based on different cable replacement criterion. Then, aerodynamic performance and wind-induce vibration of CFRP cables are studied using finite element (FE) methods based on bidirectional fluid–solid interaction theory. Wind speed and pressure distribution in the flow field around the CFRP and steel cables were studied. Aerodynamic coefficient and wind-induce vibration performance were also analyzed. Based on the aerodynamic performance and wind-induce vibration, optimal cable replacement criterion was suggested. In addition, different aerodynamic configuration design plans were proposed to mitigate the large response of CFRP cable and the optimal aerodynamic configuration were determined. This study can provide insights into the aerodynamic performance of CFRP cables and design guidance for CFRP cable in cable-supported bridges.

## Cable replacement criterion

In this study, aerodynamic performance and wind-induced response of CFRP cable were studied by comparing with steel cable. The CFRP cable was designed by replacing the longest steel cable with a diameter of 0.1 m and a length of 18.7 m in a tied arch bridge. Commonly, three cable replacement criterion, namely: stiffness equivalent criterion, strength equivalent criterion and area equivalent criterion, were adopted to replace steel cable with CFRP cable. The three cable replacement criteria are expressed as follows^31^:

For stiffness equivalent criterion, the tensile stiffness of CFRP cable is the same with the tensile stiffness of steel cable. The tensile stiffness before and after cable replacement is expressed as:

1 $$E_{{\text{C}}} A_{{\text{C}}} = E_{{\text{S}}} A_{{\text{S}}}$$

where $E_{{\text{C}}}$ and $E_{S}$ are elastic moduli of CFRP and steel, respectively; $A_{{\text{C}}}$ and $A_{S}$ are cross-sectional areas of CFRP and steel cables.

Strength equivalent criterion stipulates that the design strengths of CFRP and steel cables are equal with considering safety factor and is expressed as:

2 $$[\sigma_{C} ]A_{C} = [\sigma_{s} ]A_{s}$$

where $[\sigma ]$ denotes the allowable strength of the cable, which can be expressed as:

3 $$[\sigma ] = \frac{{f_{y} }}{S}$$

where $f_{y}$ is the ultimate strength of cable and $S$ is the design safety factor. For steel cable, the design safety factor commonly adopts 2.5. However, as CFRP is a brittle material, the design safety factor of CFRP cable is larger than that of steel cable and 3.0 is proposed as safety factor of CFRP cable by many researchers^32–34^.

Area equivalent criterion means that the steel cable is replaced with CFRP cable with identical cross-sectional areas. This cable replacement criterion can be expressed with the following equation:

4 $$A_{C} = A_{S}$$

The mechanical properties of CFRP and steel cables are listed in Table 1. The mechanical properties of CFRP cable are taken from literature^31^. Based on the Eqs. (1)–(4), the corresponding cross-sectional areas of CFRP cable calculated by stiffness equivalent, strength equivalent and area equivalent criteria are 0.126, 0.093 and 0.100 m^2^, respectively.

**Table 1:** Mechanical properties of steel and CFRP cables.

Material	Elastic modulus /(GPa)	Ultimate strengt (MPa)	Density (kg m^−3^)	Poisson’s ratio	Damping factor
Steel	201	1860	7850	0.30	0.008
CFRP	160	2400	1600	0.25	0.005

## Finite element model

In this study, computational fluid dynamics (CFD) was adopted to study the aerodynamic properties and wind-induced response of CFRP and steel cables. To construct the CFD model, the computational domain and boundary conditions, mesh generation need to be established.

### Computational domain and boundary conditions

**Figure 1:**
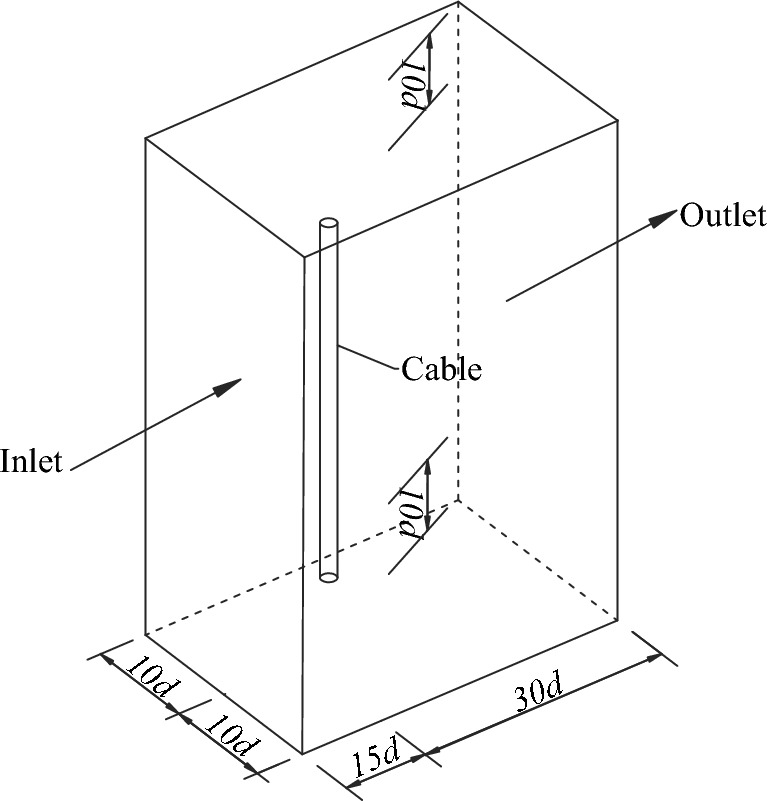
Computational domain of the flow field.

In the CFD model, the flow field was modeled as a regular hexahedron as shown in Fig. 1. The cable was placed in the flow field with consolidation at both ends. The distances from the central of the cable to the inlet and outlet were 15d and 30d (d is the diameter of the cable), respectively. The distances from the central of the cable to the left, right, top and bottom surfaces of the flow field were all 10d. The boundary conditions of inlet and outlet of the flow field were velocity-inlet and pressure-outlet, respectively. The boundary conditions of left and right surfaces of the flow field were symmetry. The boundary conditions of top and bottom surfaces of the flow field were free slip wall.

### Mesh generation

**Figure 2:**
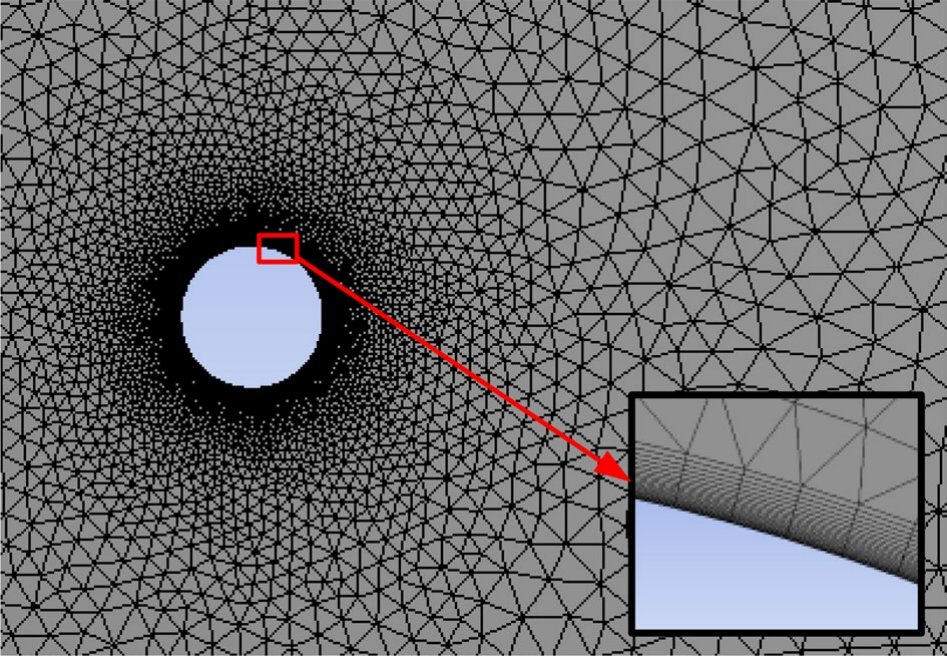
Scheme of the mesh grid.

Mesh generation determines the computation accuracy and efficiency of the CFD model. In this study, unstructured grid was meshed to balance the computation accuracy and efficiency. When fluid flows toward to the cable, the fluid flow changes dramatically near the cable and changes little away from the cable. For this reason, the mesh grid of the computation domain was much smaller and more intensive near the cable as shown in Fig. 2. While the mesh grid of the computation domain became sparser and sparser with the distance to the cable.

### Validation of the CFD model

**Figure 3:**
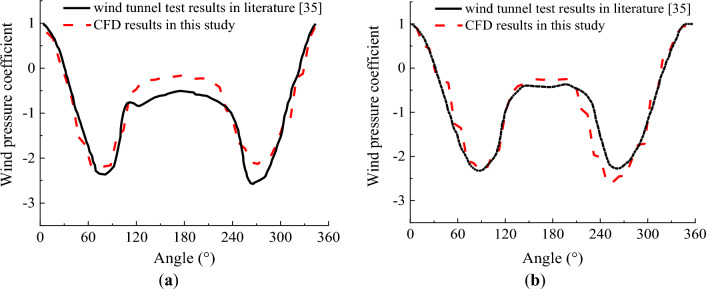
Comparison of wind pressure coefficient between CFD results in this study and test results in literatures. (**a**) Re = 3.79 × 10^5^; (**b**) Re = 4.93 × 10^5^.

**Figure 4:**
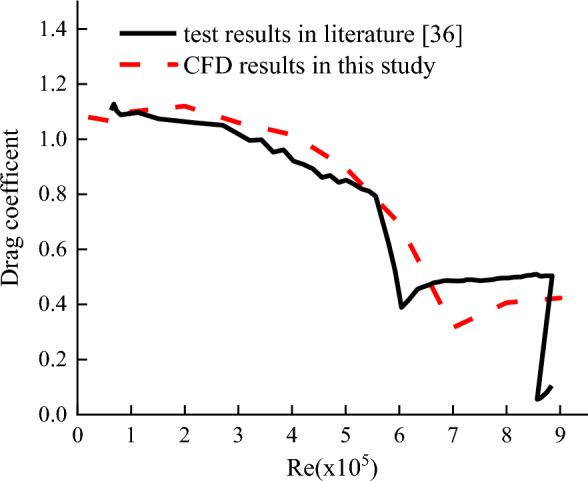
Comparison of drag coefficient between CFD results in this study and test results in literature.

To validate the CFD model, the CFD model was established and results were compared with wind tunnel test performed by Liu et al.^35^in which a circular cylinder with a length of 1200 mm and a diameter of 120 mm was applied to wind tunnel test. Test wind pressure coefficient around the cable under Reynolds number Re = 3.79 × 10^5^ and drag coefficient of the cable under different Reynolds numbers when then wind came from different angles were compared with the CFD results in Figs. 3. In addition, a circular cylinder with a height of 500 mm and a diameter of 300 mm in^36^ was also simulated with the CFD model. The simulated drag coefficients of the cylinder under different Reynolds numbers were also compared with the wind tunnel test in^36^ in Fig. 4. It is seen from Figs. 3 and 4 that the CFD results agrees well with the wind tunnel test results, indicating that the CFD model was validate to simulate the aerodynamic performance and wind induced response of the cable.

## CFD results and discussion

To study the aerodynamic and wind induced response of CFRP cable, the flow velocity distribution downstream of the cable, wind pressure before and after the cable, aerodynamic force coefficients, time history curves of cable displacement response, principal stress-load step curves of the steel and CFRP cables were simulated and compared. Then optimal cable replacement criterion was suggested based on the comparison between the steel and CFRP cables.

### Flow velocity distribution downstream of the cable

**Figure 5:**
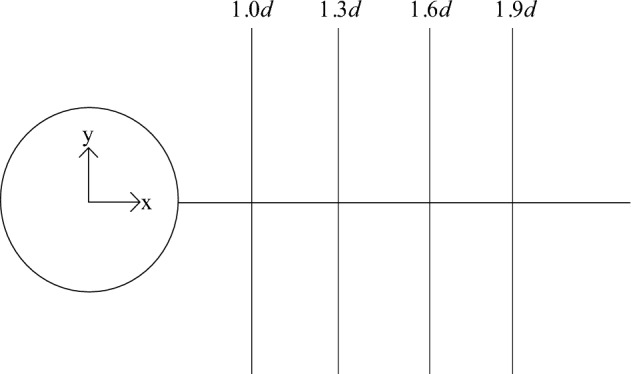
Arrangement of flow velocity sampling.

Flow velocity distribution downstream of the cable is a vital factor to reflect the aerodynamic performance of the cable. To better investigate the wake flow characteristics downstream of the cable, the XY plane at midspan of the cable was selected as the analysis plane. The centerline of the wake at y/d = 0 was selected as the targeted line to extract the flow velocity distributions. The specific arrangement of flow velocity sampling is shown in Fig. 5.

**Figure 6:**
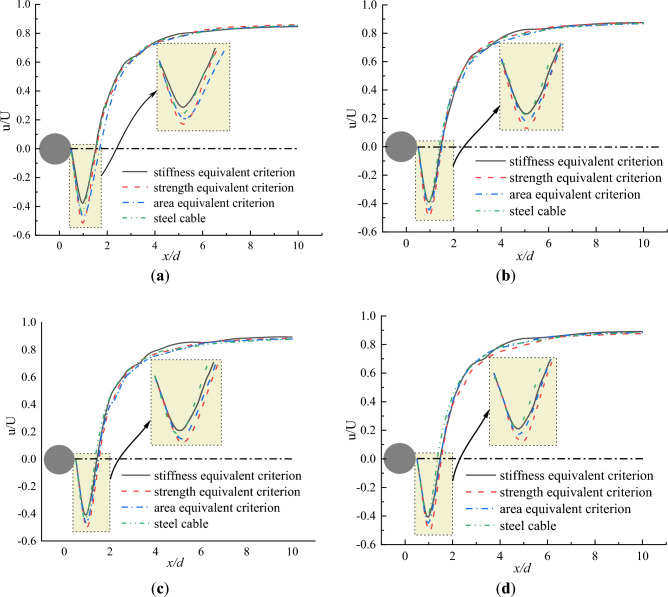
Flow velocity distributions downstream of the steel and CFRP cable. (**a**) U = 10 m/s; (**b**) U = 15 m/s; (**c**) U = 20 m/s; (**d**) U = 25 m/s.

Figure 6 shows the dimensionless mean velocity distributions at the wake centerline behind the CFRP and steel cables under different initial wind speeds of 10, 15, 20 and 25 m/s. In Fig. 6, “u” is the streamwise mean velocity, “U” is the initial wind speed. It is seen from Fig. 6 that there existed a negative velocity with a “V” shaped distribution that first decreased then increased within the range of about 0.5–2.0d behind both steel and CFRP cables. This indicates that vortices exist behind the cables, leading to a fluid flow opposite mainstream direction. As the distance increased, the velocity transitioned from negative to positive and gradually increased to a stable value at the location of about x = 6d. Besides, under different initial wind speeds, the reverse flow zone behind the steel cable existed in a range of 0.5–1.5d. However, the reverse flow zones for CFRP cables under stiffness, strength and area equivalent criteria were 0.5–1.53d, 0.5–1.58d, 0.5–1.54d, respectively. It was obvious that the reverse flow regions for CFRP cables were larger than that of steel cable. This was attributed to the relative low density of CFRP cable. As the density of CFRP cable was much lower than that of steel cable, CFRP cables had poorer dynamic similitude with air compared to steel cables. Additionally, the lower inertial force of CFRP cable was less effective at suppressing pulsations in the flow field. This results in more pronounced vortex shedding behind CFRP cables, leading to larger reverse flow region for CFRP cable compared to steel cable.

**Figure 7:**
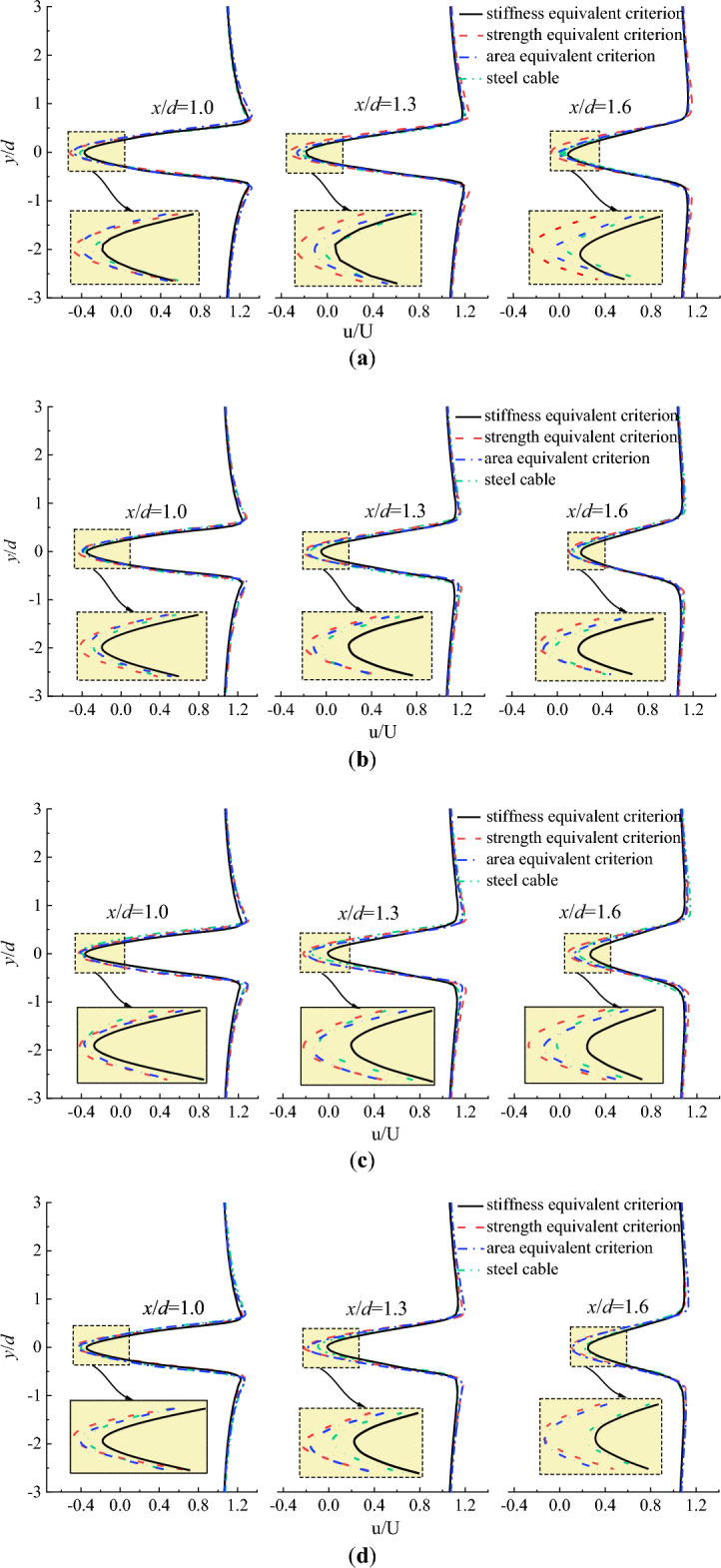
Flow velocity distribution perpendicular to the initial wind flow at different locations. (**a**) U = 10 m/s; (**b**) U = 15 m/s; (**c**) U = 20 m/s; (**d**) U = 25 m/s.

Figure 7 plots the flow velocity distribution perpendicular to the initial wind flow at different locations. As the reverse flow existed with in the region of 0.5–1.8d, the observation locations of the flow velocity distribution perpendicular to the initial wind flow were set as x/d = 1.0d, 1.3d and 1.6d as shown in Fig. 6. It can be seen from Fig. 7 that the maximum negative flow velocity of CFRP cable based on stiffness equivalent criterion was smaller than that of steel cable at any locations. However, the maximum negative flow velocity of steel cable was smaller than that of CFRP cable based on area equivalent criterion. The maximum negative flow velocity of CFRP cable based on strength equivalent criterion was the largest.

From the perspective of local stability of cable, the enlargement of the reverse flow region increases the instability of the airflow. In engineering practice, the air flow behind the cable is expected to be stable and the reverse region of the flow is expected to be small to reduce disturbance of the cable. In addition, the negative velocity in the reverse flow region is also an index to indicate the local stability of the flow behind the cable. The larger the magnitude of the negative velocity, the less stable of the air flow behind the cable. For this reason, the stiffness equivalent criterion is proposed when steel cable is replaced with CFRP cable.

### Wind pressure difference before and after the cable

**Figure 8:**
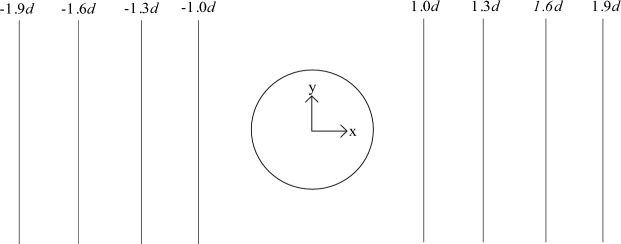
Observation locations of wind pressure before and after cable.

When wind flows past the cable, the wind flow velocity decreases due to obstruction of the cable, leading to increased wind pressure. On the other hand, the wind pressure decreases at the rear side of the cable. Vortex flow regions are thus formed at the rear side of the cable due to flow separation, resulting in a pressure difference between the front and rear sides of the cable. The pressure difference can easily excite great vibration of the cable. Hence, wind pressures before and after the cable are vital to evaluate the aerodynamic performance of the cable. In this study, four points of *x* = − 1.0*d*, − 1.3*d*, -1.6*d* and − 1.9*d* before the cable and four points of *x* = 1.0*d*, 1.3*d*, 1.6*d* and 1.9*d* after the cable were set as the observation locations of the wind pressure as shown in Fig. 8. To intuitively compare the wind pressure data variations, the wind pressure was nondimensionalized to obtain the pressure coefficient as follows^30^:

5 $$P_{i} = \frac{{p_{i} - p_{\infty } }}{{0.5\rho U_{\infty }^{2} }}$$

where $P_{i}$ is the wind pressure coefficient at the targeted location, $p_{i}$ is the wind pressure at the targeted location,$p_{\infty }$ is the wind pressure at the entrance of wind flow, $\rho$ is the air density and $U_{\infty }$ is the initial wind speed at the entrance of wind flow.

**Figure 9:**
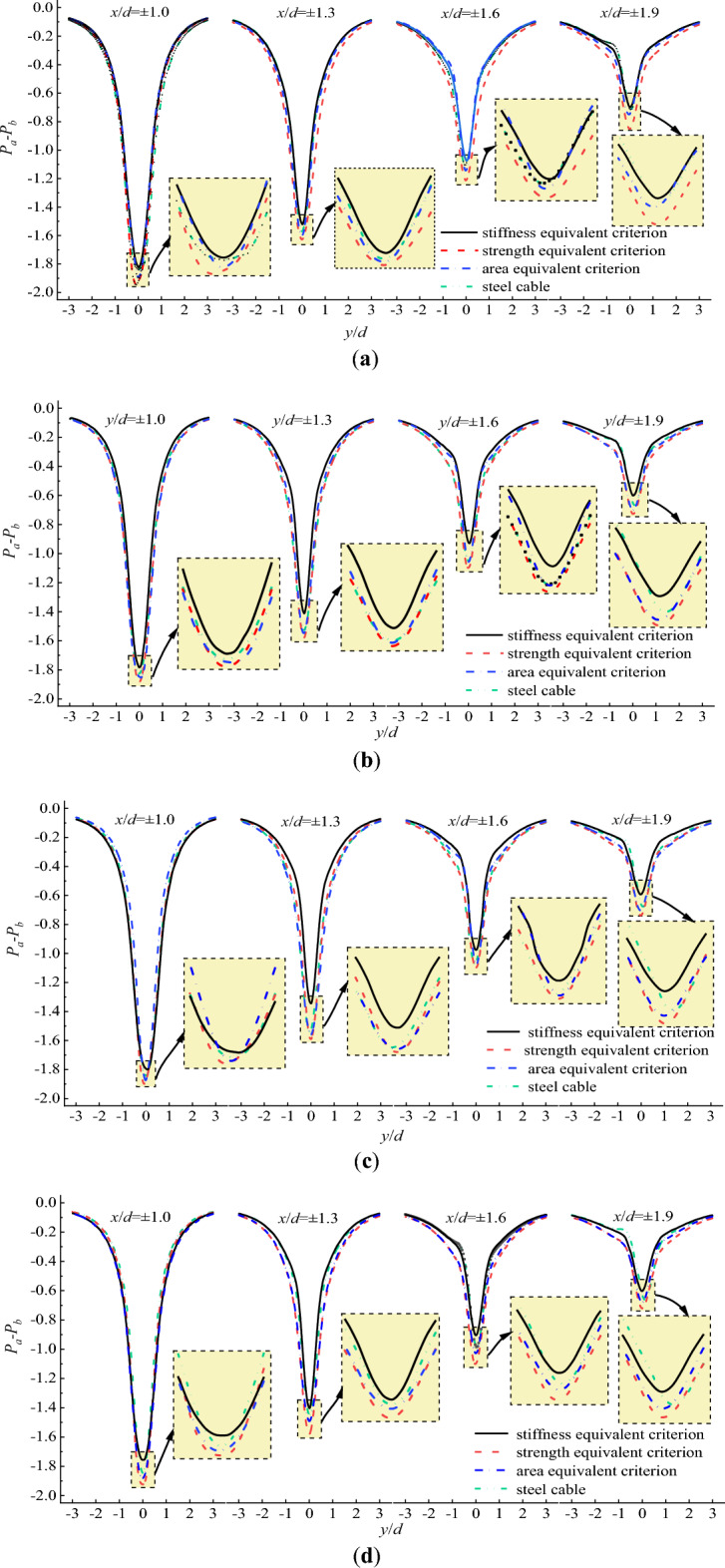
Wind pressure coefficient differences before and after CFRP and steel cables under different initial wind velocities. (**a**) U = 10 m/s; (**b**) U = 15 m/s; (**c**) U = 20 m/s; (**d**) U = 25 m/s.

The nondimensionalized wind pressure difference $\Delta P_{i}$ can thus be expressed as:

6 $$\Delta P_{i} = P_{ai} - P_{bi}$$

where $P_{ai}$ and $P_{bi}$ are nondimensionalized wind pressures after and before the cable at the targeted location. Based on Eq. (6), the nondimensionalized wind pressures difference at the observation locations can be calculated and plotted in Fig. 9.

It is seen from Fig. 9 that the distribution of wind pressure coefficient difference was similar with that of dimensionless mean velocity distribution behind the cable with a “V” shape. The wind pressure coefficient differences under different initial wind velocities were almost the same. This is because the influence of initial velocity on the wind pressure coefficient was excluded in Eq. (5). Considering the influence of initial wind velocity, the wind pressure difference under higher initial wind velocity was much larger than that under lower initial wind velocity. However, the wind pressure coefficient difference decreased with the distance from the observation location to the cable body for both CFRP and steel cables. In addition, regardless of the initial wind velocity and the observation location, the wind pressure coefficient difference for CFRP cable based on strength equivalent criterion was the largest, followed by those for CFRP cable based on area equivalent criterion and steel cable in order. The wind pressure coefficient difference for CFRP cable based on stiffness equivalent was the smallest among all the cables. This was because the wind pressure before and after the cable mainly depended on the cross-sectional area of the cable. A lager cross-sectional area resulted in a larger wind pressure difference. Though steel cable and CFRP cable based on area equivalent criterion had the same cross-sectional area, the CFRP cable had a much larger deformation under wind load because of the much smaller stiffness. Thus, the wind pressure behind the cable was smaller than that of steel cable and the wind pressure difference of CFRP cable under area equivalent criterion was larger than that of steel cable. Moreover, the differences among the four cables were not obvious when the initial wind velocity was small. However, the differences among the four cables became more and more obvious.

For engineering practice, the wind pressure difference before and after the cable can essentially be seen as an excitation on the cable. A larger wind pressure difference means a larger excitation and would cause larger amplitude of the cable. To this end, the stiffness equivalent criterion is suggested for CFRP cable to replace steel cable for decreasing the wind pressure difference and amplitude of the cable.

### Drag and lift coefficients of the cable

**Figure 10:**
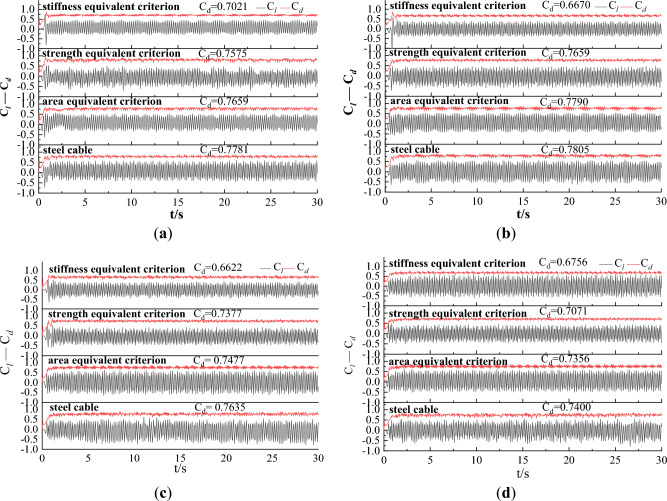
Time history of drag and lift coefficients for steel and CFRP cables under different wind velocities. (**a**) U = 10 m/s; (**b**) U = 15 m/s; (**c**) U = 20 m/s; (**d**) U = 25 m/s.

When wind flows past the cable, drag force $F_{d}$ is generated due to the uneven wind pressure around the cable. A lift force $F_{l}$ is also generated due to the unequal pressure distribution between the top and bottom surfaces of the cable. The drag and lift forces were obtained from the CFD model, and drag and lift coefficients can be calculated with the following equations^37^:

7 $$C_{d} = \frac{{F_{d} }}{{0.5\rho AU^{2} }}$$

8 $$C_{l} = \frac{{F_{l} }}{{0.5\rho AU^{2} }}$$

where $C_{d}$ and $C_{l}$ are the drag and lift coefficients, respectively; $A$ is the cross-sectional area of the cable. Based on Eqs. (7) and (8), time history of drag and lift coefficients for steel and CFRP cables under different wind velocities were calculated and plotted in Fig. 10.

It is seen from Fig. 10 that drag and lift coefficients eventually stabilized at nearly constant values. The lift coefficient fluctuated around 0 and the average of the lift coefficients was nearly 0 due to the symmetric nature of the vortex shedding. The average drag effect under each condition was also labeled in Fig. 10. The average drag coefficient showed little difference under different initial wind velocities for both steel and CFRP cables. However, the CFRP cable based on stiffness equivalent criterion exhibited the minimum average drag coefficient. The average drag coefficient of CFRP cable based on strength equivalent criterion was larger than that of CFRP cable based on stiffness equivalent criterion, but smaller than that of CFRP cable based on area equivalent criterion. Steel cable exhibited the maximum average drag coefficient. This phenomenon was independent of the initial wind velocity.

**Figure 11:**
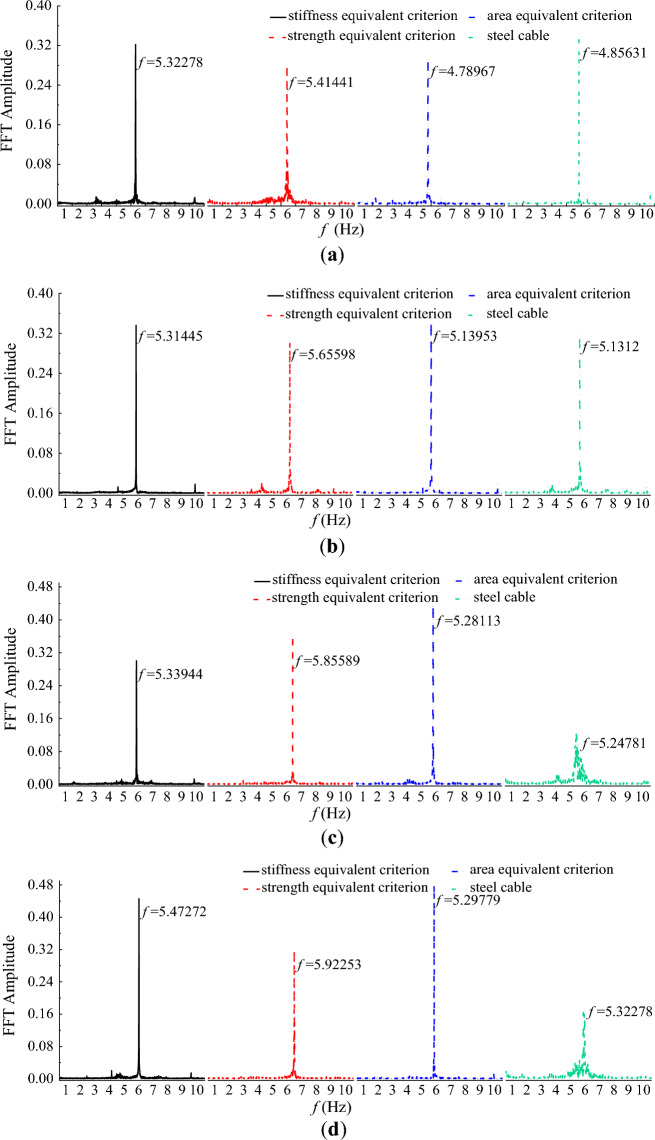
Frequency spectrum of the lift coefficient for different cables under different wind velocity. (**a**) U = 10 m/s; (**b**) U = 15 m/s; (**c**) U = 20 m/s; (**d**) U = 25 m/s.

The frequency spectrum of the lift coefficient provides insights into the unsteady aerodynamic forces acting on a body. The frequency of the lift coefficient in essence is the vortex shedding frequency. In this study, the frequency spectrum of the lift coefficient was obtained based on Fast Fourier Transform (FFT) and plotted in Fig. 11. It is clearly seen from Fig. 11 that the vortex shedding frequency increases with the initial wind velocity. This is because the wind can be regarded as excitation of the cable. A larger wind velocity means a larger excitation and results in a faster vibration of the cable and more frequent vortex shedding phenomenon. In addition, the vortex shedding frequency of the steel cable was larger than that of CFRP cable. For CFRP cable, the vortex shedding frequency based on area equivalent criterion was larger than that based on strength equivalent criterion. The vortex shedding frequency of CFRP cable based on stiffness equivalent criterion was the smallest among all the cables.

In engineering practice, a smaller drag coefficient and a smaller vortex shedding frequency result in a more stable vibration of the cable. Hence, the stiffness equivalent criterion was suggested for replacing steel cable with CFRP cable from the perspective of drag coefficient and vortex shedding frequency.

### Vibration displacement time history of the cable

**Figure 12:**
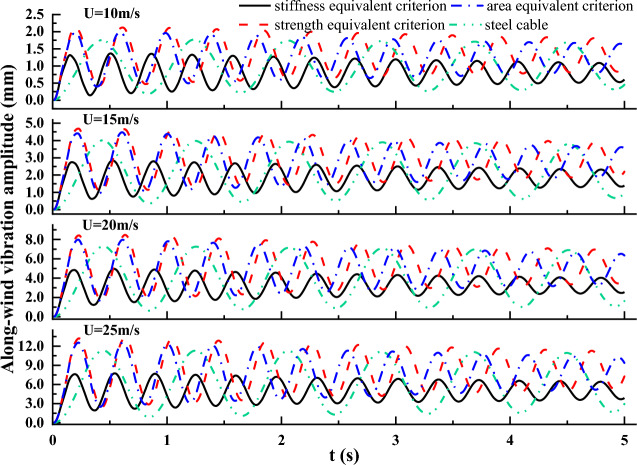
Along-wind vibration displacement time histories of steel and CFRP cable.

**Figure 13:**
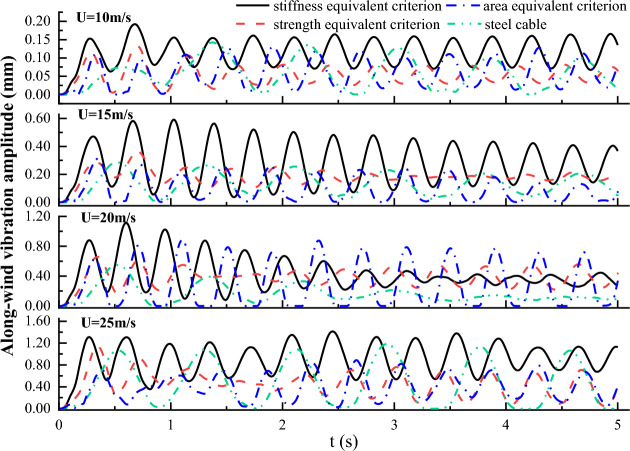
Cross-wind vibration displacement time histories of steel and CFRP cable.

Under the excitation of wind load, cables in cable supported bridges are prone to dynamic wind-induced vibration. Figures 12 and 13 plot the displacement time history of the cable vibration in along-wind and across-wind directions, respectively. Figures 12 and 13 show that vibration displacements of steel and CFRP cables all increased with the initial wind velocity both in along-wind and across-wind directions. In addition, the vibration responses in along-wind direction exceeded significantly that in across-wind direction. This was mainly because the along-wind vibration was attributed to the drag force resonance, while the across-wind vibration was mainly contributed by the transverse vortex shedding effects, which was much weaker than the drag force. Moreover, the CFRP cable based on strength equivalent criterion exhibited the largest along-wind direction vibration amplitude, followed by CFRP cable based on area equivalent criterion, then steel cable, and CFRP cable based on stiffness equivalent exhibited the smallest vibration amplitude. This was mainly because the along-wind vibration displacement was closely related to the flexural stiffness of the cable. Among the four cables, the magnitude of the flexural stiffness followed the sequence CFRP cable based on stiffness equivalent criterion, steel cable, CFRP cable based on area equivalent criterion, CFRP cable based on strength equivalent criterion from highest to lowest. However, there was no obvious relationship or pattern among the cross-wind displacement magnitudes of steel cable and CFRP cables based on different cable replacement criteria. This was mainly due to the irregular vortex shedding.

**Figure 14:**
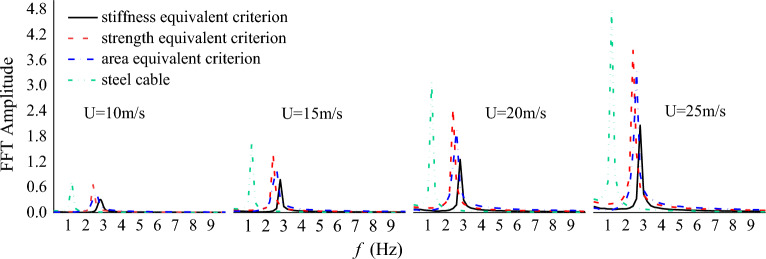
Frequency spectrum of the along-wind vibration displacement for different cables under different wind velocity.

**Figure 15:**
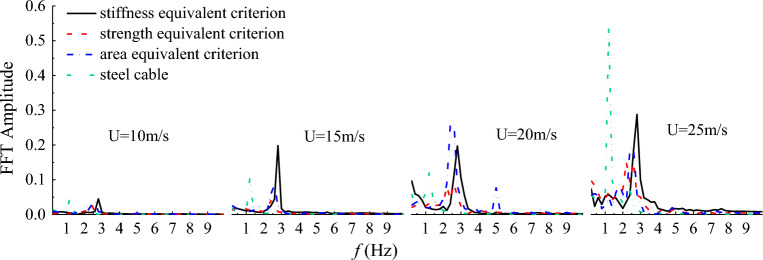
Frequency spectrum of the cross-wind vibration displacement for different cables under different wind velocity.

The frequency spectrums of the along-wind and cross-wind vibration displacements for steel and CFRP cables under different initial wind velocity were also derived based on FFT and plotted in Figs. 14 and 15. It is seen from Figs. 14 and 15 that the vibration frequencies in along and cross-wind directions show identical pattern for both steel and CFRP cables. First, the vibration frequencies of steel and CFRP cables all increased with the initial wind velocity. Besides, regardless of the initial wind velocity, the CFRP cable based on stiffness equivalent criterion exhibited the largest along and cross-wind vibration frequencies, followed by CFRP cables based on area and strength equivalent criteria, and the steel cable exhibited the lowest along and cross-wind vibration frequencies. Despite higher wind-induced vibration frequency of cables affects cable fatigue performance, replacing steel cables with CFRP cables is still feasible considering the excellent fatigue resistance of FRP materials. Considering CFRP cable based on stiffness equivalent criterion exhibited the lowest along-wind vibration displacement magnitude among all the cables, it is suggested to adopt the stiffness equivalent criterion when replacing steel cables with CFRP ones.

### Principal stress-loading step curve

**Figure 16:**
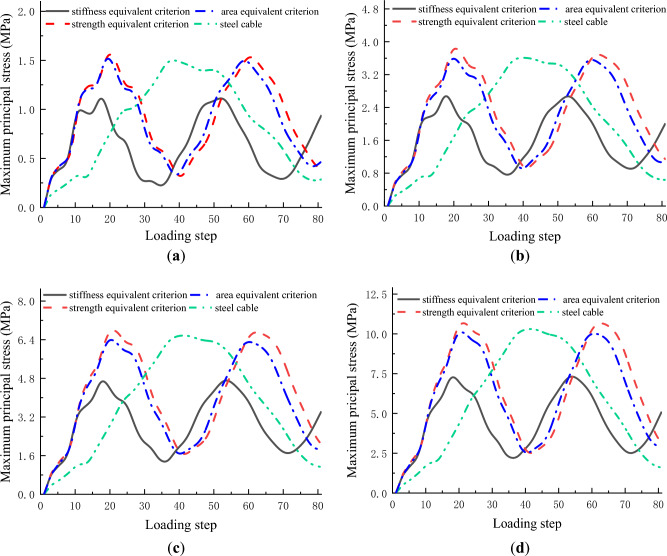
Evolution curves of maximum principal stress with loading steps for steel and CFRP cables under different wind velocities.

Figure 16 plots the evolution curves of maximum principal stress with loading steps for steel and CFRP cables. As steel cable and CFRP cables in this study exhibited different wind-induced vibration frequencies, the loading steps corresponding to the maximum stress in the first cycle for the steel cable was taken as the benchmark. The principal stress evolution curves at this loading step were extracted for both steel and CFRP cables. It is seen from Fig. 16 that the maximum principal stresses increased with the initial wind velocity for both steel and CFRP cables. In addition, the maximum principal stresses of steel cable, CFRP cables based on area and strength equivalent criteria showed little difference. However, the maximum principal stress of CFRP cable based on stiffness equivalent was much lower than those of steel cable and CFRP cables based on area and strength equivalent criteria. A smaller maximum principal stress means a better fatigue performance. From this perspective, the stiffness equivalent criterion is suggested for CFRP cable to replace steel cable.

From the above analysis, it is seen that CFRP cables based on strength and area equivalent criteria exhibited poorer aerodynamic performance than steel cable in most cases. While CFRP cable based on stiffness equivalent criterion performed superior aerodynamic performance compared with CFRP cables based on strength and area criteria. Compared with steel cable, CFRP cable based on stiffness equivalent criterion exhibited lower negative flow velocity downstream the cable, smaller wind pressure differentials before and after the cable, reduced along-wind vibration displacement and lower principal stress magnitudes. Nevertheless, the reverse flow zone, vortex shedding frequency and wind-induced vibration frequency of CFRP cable based on stiffness equivalent criterion were still larger than those of steel cable. Considering the aerodynamic performance and wind-induced response of CFRP cables based on different cable replacement criteria, the stiffness equivalent criterion is suggested to replace steel cable. However, CFRP cable based on stiffness equivalent criterion still needs a optimal design to achieve aerodynamic performance and wind-induced vibration response competitive with steel cable before practical application.

## Aerodynamic performance optimization of CFRP cable

As described above, CFRP cable based on stiffness equivalent criterion is suggested to replace steel cable from the perspective of aerodynamic performance as CFRP cable based on stiffness equivalent criterion performes superior aerodynamic performance than CFRP cables based on strength and area equivalent criteria. However, CFRP cable based on stiffness equivalent still needs to be optimized as it exhibits inferior aerodynamic performance compared with steel cable in some aspects such as the reverse flow zone, vortex shedding frequency and wind-induced vibration frequency. Hence, the main objective of optimal design for CFRP cable based on stiffness equivalent criterion is to endow it with exhibit competitive aerodynamic performance compared to steel cable.The most contemporary methods for aerodynamic performance optimization for cables involve altering the cable surface, including adding groove textures, dimple concavities, and ribbed protrusions along the cable axis. In this study, the effects of engraving symmetric and asymmetric V-shaped grooves on the cable surface were studied. In addition, aerodynamic effects of helical and vertical V-shaped grooves were also discussed. Then, the optimal surface modification was proposed.

### Geometry of the V-shaped groove

**Figure 17:**
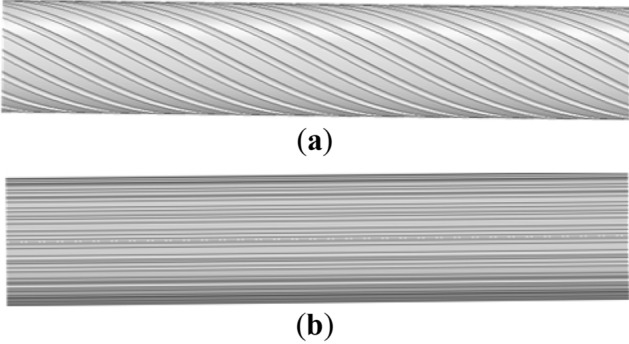
Helical and vertical grooves. (**a**) Helical grooves; (**b**) Vertical grooves.

**Figure 18:**
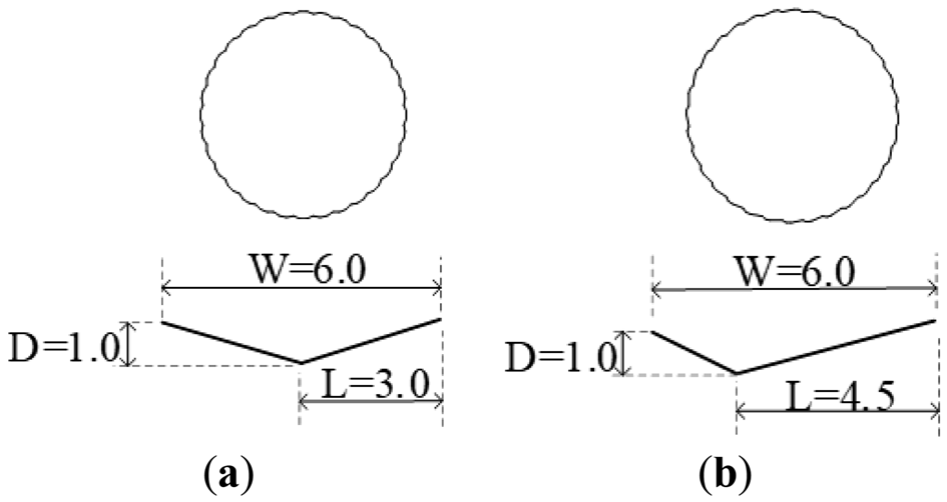
Dimensions of symmetric and asymmetric grooves (unit: mm). (**a**) Symmetric groove; (**b**) Asymmetric groove.

As described above, helical and vertical V-shaped grooves were involved to study the effects of geometry on aerodynamic performance optimization. The helical and vertical grooves are shown in Fig. 17. Dimensions of symmetric and asymmetric V-shaped grooves are plotted in Fig. 18. The symmetric and asymmetric V-shaped grooves had identical width of 6 mm and height of 1 mm. Then, vertical symmetric V-shaped groove (VS groove) and vertical asymmetric V-shaped groove (VA) were compared to study the effects of symmetric and asymmetric V-shaped grooves on the aerodynamic performance of CFRP cables. Helical symmetric V-shaped groove (HS groove) and VS groove were compared to assess the influence of helical and vertical V-shaped grooves on the aerodynamic performance. For fair of comparison, 32 V-shaped grooves were uniformly distributed around the circumference for VS, VA, and HS grooves.

Since CFRP cable based on stiffness equivalent criterion exhibited deficiencies in reverse flow zone, vortex shedding frequency and wind-induced vibration frequency compared to steel cable, the aerodynamic optimization of CFRP cables will focus on these aspects. According to the analysis in “*CFD results and discussion*” section, higher initial wind velocity only increased the magnitude of the aerodynamic response, it did not affect the qualitative trends between the cables. Therefore, only the aerodynamic responses of difference cables under initial wind velocity of 20 m/s were presented in this section.

### Flow velocity distribution downstream of the CFRP cable with different surface textures

**Figure 19:**
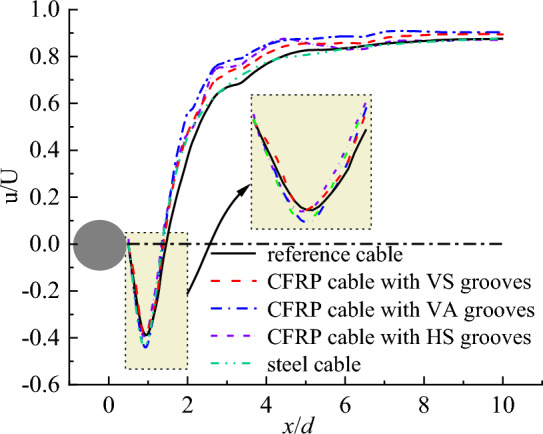
Flow velocity distributions downstream for steel cable and CFRP cable with different surface textures.

Figure 19 plots the flow velocity distributions downstream of CFRP cable with different surface textures. For the convenience of evaluating the optimizing effect, the the flow velocity distributions downstream of steel cable is also plotted in Fig. 19. It is seen from Fig. 19 that engrooving V shaped grooves had a beneficial effect on decreasing the reverse flow zone behind the cable regardless of the groove texture. This was because the grooves provided channels for partial air to escape, reducing the upstream backflow and diminishing the reverse flow zone. In addition, for different groove textures, the reverse flow zone of CFRP cable with VA grooves was larger than that of CFRP cable with VS grooves. CFRP cable with HS grooves exhibited the smallest reverse flow region. Compared with steel cable, CFRP cable based on equivalent criterion with HS grooves even exhibited smaller reverse flow zone behind the cable compare with steel cable. This was because the symmetric disturbances generated by the VS grooves caused approximately equivalent boundary layer separation delays on both sides of the cable, thereby reducing the total area and scope of the reverse flow region. However, VA grooves caused unequal boundary layer separation delays and resulted in asymmetric disturbances, thus exhibiting poor reduction effects on reverse flow region. In addition, the HS grooves generated spiral disturbances along the cable length which can act on the boundary layer uniformly and continuously. While the disturbances were only generated in the localized regions around the VS grooves. Thus, HS grooves were more conducive to reduce the scope of the separation region and reverse flow region.

**Figure 20:**
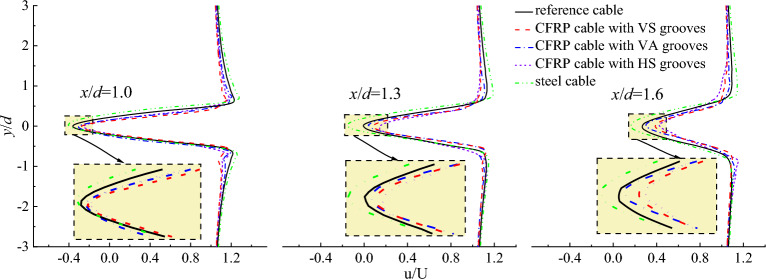
Flow velocity distribution perpendicular to the initial wind flow for steel cable and CFRP cable with different surface textures.

Figure 20 shows the flow velocity distribution perpendicular to initial wind flow for CFRP cable with different surface textures. It is seen from Fig. 20 that with V-shaped grooves, CFRP cable exhibited smaller maximum negative flow velocity than steel cable and reference cable. For different groove textures, the CFRP cable with HS grooves exhibited the lowest maximum negative flow velocity, followed by CFRP cable with VS grooves. While CFRP cable with VA grooves exhibited largest maximum negative flow velocity. Moreover, this difference became larger with the increase in the longitudinal distance along the initial wind direction. The difference can also be explained by the disturbances generated by different groove textures.

### Drag and lift coefficients of CFRP cable with different surface textures

**Figure 21:**
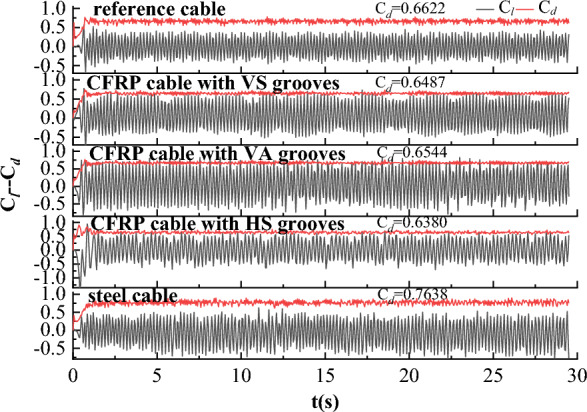
Time histories of drag and lift coefficients for steel cable and CFRP cable with different surface textures.

As discussed in “Drag and lift coefficients of the cable” section, the drag coefficient was decreased when steel cable was replaced with CFRP cable. However, the vortex shedding frequency of CFRP cable was inferior to that of steel cable. To assess the influence of V-shaped grooves on vortex shedding frequency, the time histories of drag and lift coefficients for steel cable and CFRP cable with different surface textures were also plotted in Fig. 21. It is clearly seen that engrooving V-shaped grooves further decrease the drag coefficient of the CFRP cable. Besides, the HS grooves achieved the best effect in decreasing the drag coefficient. Compared with steel cable, drag cofficient of CFRP cable with HS grooves decreased by 16.5%. VS grooves exhibited the medium effect and VA grooves achieved the minimum drag coefficient decrease among the three V-shaped groove textures. This was because grooves enabled partial boundary flow to drain through, reducing the accumulation of low-speed fluid parcels on the surface and decreasing the pressure drag component contributing to drag force. In addition, localized acceleration regions were formed around the groove openings, and the existence of these low-drag zones also contributed to the overall reduction of the drag coefficient. Compared with VS grooves, spiral disturbances generated by HS grooves were more beneficial for improving the attached flow on the cable surface and shifting the boundary layer outwards, thus resulting in a greater reduction of the low-speed recirculation region that contributed to drag generation. Compared with VA grooves, VS grooves resulted in symmetric boundary layer separation and a much larger low-speed recirculation region, thus exhibiting a smaller drag coefficient.

**Figure 22:**
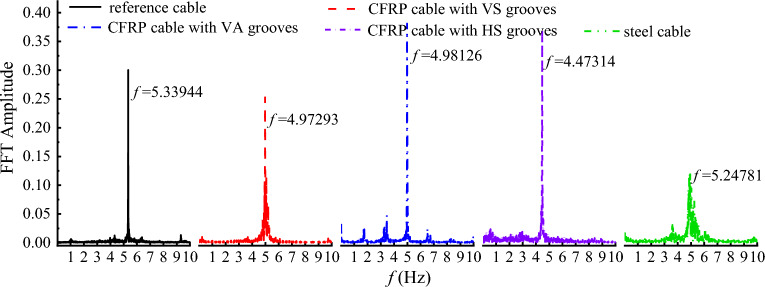
Frequency spectrum of the lift coefficient for steel cable and CFRP cable with different surface textures.

Based on the time-history of lift coefficient, the frequency spectrum of lift coefficients for steel and CFRP cable with different surface textures were derived and plotted in Fig. 22. Without grooves, the vortex shedding frequency of CFRP cable was larger than that of steel cable. However, with V-shaped grooves, the vortex shedding frequency was obviously decreased. The vortex shedding of CFRP cable with V-shaped grooves was much smaller than that of steel cable. Moreover, it is seen from Fig. 19 that the vortex shedding frequencies of CFRP cables with VS and VA grooves were almost the same. While vortex shedding frequency of CFRP cable with HS grooves was much smaller than that of CFRP cable with VS and VA grooves, indicating that HS grooves had the best vortex shedding decrease effect.

### Wind-induced vibration of CFRP cables with different surface textures

**Figure 23:**
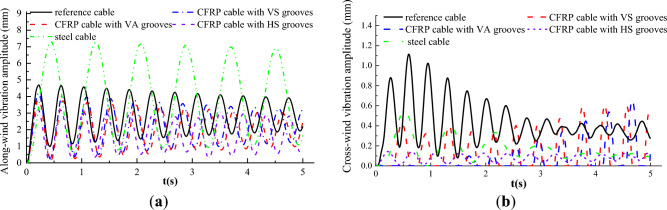
Wind-induced vibration displacement time histories of steel cable and CFRP cable with different surface textures. (**a**) Along-wind vibration; (**b**) Cross-wind vibration.

**Figure 24:**
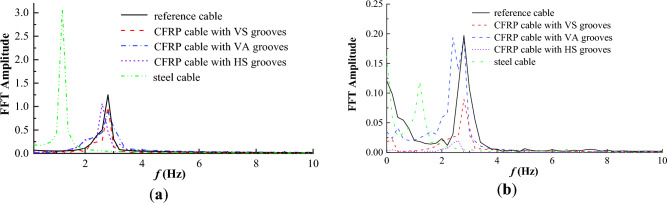
Frequency spectrum of wind-induced vibration displacement for steel cable and CFRP cable with different surface textures. (**a**) Along-wind vibration; (**b**) Cross-wind vibration.

As described in “*Vibration displacement time history of the cable*” section, the CFRP cable exhibited a higher wind-induced vibration frequency. To evaluate the effects of different V-shaped grooves on decreasing the wind-induced vibration frequency, the along and across-wind vibration displacements of steel cable and CFRP cable with different surface textures are plotted in Fig. 23 and vibration frequencies for steel cable and CFRP cables with different V-shaped grooves are obtained and plotted in Fig. 24 on the basis of the vibration displacement time history. It is seen from Fig. 23 that the along and cross-wind vibration displacements were obviously decreased compared with reference and steel cables by engrooving V-shaped grooves. Besides, the decrease effect for HS grooves was still the best, followed by VS grooves and VA grooves in order. For along wind vibration displacement, it was greatly affected by the flow passed by the cable body. By engrooving V-shaped grooves, the flow could escape from the groove boundaries and the low-speed flow region was decreased, thus weakening the wind excitation forces. HS grooves had the best effect in decreasing the low-speed flow region, thus exhibiting the best effect in decreasing the along-wind vibration displacement. For across-wind vibration, it was mainly induced by vortex shedding behind the cable. As HS grooves exhibited the best vortex shedding decrease effect, CFRP cable with HS grooves performed the lowest across-wind vibration displacement.

Though engrooving V-shaped grooves could decrease the wind-induced vibration displacement of CFRP cable, the wind-induced vibration frequency changed little as shown in Fig. 24, indicating that the V-shaped grooves had little effect in decreasing the wind-induced vibration frequency. This is mainly because the vibration frequency of CFRP cable attributes to the cable mass, length and elastic modulus. The outer wind excitation does not change the vibration frequency. A larger vibration frequency imply a poorer vibration fatigue performance. To address this issue, vibration reduction measures such as semi-active or active dampers may be effective solutions^38–42^. In addition, as previously discussed, engrooving V-shaped grooves could sifnificantly reduce the along and across-wind vibration amplitudes, which is also benficial for the fatigue resistance of the cable. Moreover, as CFRP cable inherently has better fatigue resistance compared to steel cable. Taking into account all the above factors, the fatigue issues of CFRP able based on stiffness equivalent criterion caused by high wind-induced vibration frequency can be adequately resolved in practice to endow CFRP cable with fatigue resistance that is competitive to steel cable.

From the above analysis, it is concluded that engrooving V-shaped groves could effectively improve the aerodynamic performance and wind-induced vibration properties of CFRP cable compared to the reference CFRP cable. Particularly, by engrooving HS grooves, both the reverse flow region and the vortex shedding frequency of CFRP cable, which were inferior to those of steel cable before surface optimization, were reduced to levels even lower than those of steel cables. Though wind-induced vibration frequency of CFRP cable based on stiffness equivalent criterion was not apparently decreased, the wind-induced vibration amplitude was effectively reduced and fatigue issues of CFRP cable based on stiffness equivalent criterion caused by the high wind-induced vibration frequency can be controlled by means of vibration reduction measures. In addition, symmetric V-shaped grooves outperformed asymmetric patterns in improving aerodynamic performance and wind-induced vibration properties. Moreover, helical V-shaped grooves exhibited a more excellent effect of improving aerodynamic performance to vertical grooves. Thus, HS grooves is recommended to modify surface of CFRP cable for a better aerodynamic performance and wind-induced vibration properties.

## Life-cycle cost analysis

When CFRP cable based on stiffness equivalent criterion is adopted to replace steel cable, the usage of CFRP volume will be 1.26 times that of steel cables as decribed in “*Cable Replacement Criterion*” Secrtion. In addition, the material price per unit mass of CFRP cable is almost 10 times that of steel cable^43^. As such, despite the density of CFRP acble being only around 1/5 of steel cable as decribed in Table 1, the material cost of using CFRP cables will be 2.6 times that of using steel cables. However, assuming a survice life of 100 years of the cable-supported bridges, during the service period the steel cables would need replacement at least once due to corrosion and fatigue issues, whereas the CFRP cables would basically require no replacement throughout the entire service life^43,44^. Thus, the replacement of steel cable further increases the life-cycle material cost of steel cable-supported bridges. Apart from material costs, cable replacement would also incur additional construction costs. In addition, with continuous technological advances and increased adoption, the costs of CFRP materials will steadily decrease. Moreover, considering that many steel cable-stayed bridges require more than one cable replacement projects over their whole life cycle, employing CFRP cables based on stiffness equivalent criterion is economically viable for practical engineering applications^43,44^.

## Conclusions

In this study, the aerodynamic performance and wind-induced vibration properties of CFRP cables were studied and compared with those of steel cable. The CFRP cable were designed by replacing the steel cable based on stiffness equivalent, area equivalent and strength equivalent criteria. The flow velocity distribution downstream of the cable, wind pressure difference before and after the cable, drag and lift coefficients, wind-induced vibration displacement and principal stress were analyzed by CFD model. Then, the aerodynamic performance and wind-induced vibration properties of CFRP cable were optimized by engrooving VS, VA and HS grooves. The effects of different groove textures were compared and the best surface modification plan was proposed. Based on this study, the following conclusions can be drawn:

1. For both steel and CFRP cables, the patterns of aerodynamic response and wind-induced vibration properties were similar under different wind velocities, while the quantitative values increased proportionally with the speed.
2. Compared with steel cable, CFRP cable exhibited inferior aerodynamic response and wind-induced vibration properties in most case except for the drag coefficient. The reverse flow zone, negative flow velocity downstream the cable, wind pressure difference before and after the cable, vortex shedding frequency, wind-induced vibration displacement and principal stress of CFRP cables based on strength and area equivalent criteria were markedly inferior to steel cable. However, CFRP cable based on stiffness equivalent criterion exhibited lower negative flow velocity downstream the cable, smaller wind pressure differentials before and after the cable, reduced along-wind vibration displacement and lower principal stress magnitudes than steel cable. Thus, the stiffness equivalent criterion was suggested to replace steel cable with CFRP cable from the perspective of aerodynamic response and wind-induced vibration properties.
3. Though CFRP cable based on stiffness equivalent criterion was suggested to replace steel cable, it exhibited larger reverse flow zone, vortex shedding frequency and wind-induced vibration frequency than steel cable. Engrooving V-shaped grooves on the cable surface could effectively improve these performances to levels that were competitive with steel cable as V-shaped grooves enabled partial boundary flow to drain through and improved the wake flow conditions behind the cable.
4. Compared with asymmetric V-shaped grooves, symmetric grooves performed better effect in improving aerodynamic performance and wind-induced vibration properties as symmetric grooves generated symmetric disturbances and caused approximately equivalent boundary layer separation delays. In addition, helical grooves outperformed vertical grooves in improving aerodynamic performance and wind-induced vibration properties as helical grooves generated spiral disturbances along the cable length which can act on the boundary layer uniformly and continuously. Thus, HS grooves was proposed to be engrooved on the CFRP cable based on stiffness equivalent criterion to replace steel cable in engineering practice.

## Data Availability

The datasets used and analyzed during the current study are available from the corresponding author on reasonable request.
